# Different Neural Information Flows Affected by Activity Patterns for Action and Verb Generation

**DOI:** 10.3389/fpsyg.2022.802756

**Published:** 2022-03-24

**Authors:** Zijian Wang, Zuo Zhang, Yaoru Sun

**Affiliations:** ^1^School of Computer Science and Technology, Donghua University, Shanghai, China; ^2^Social, Genetic and Developmental Psychiatry Centre, Institute of Psychiatry, Psychology & Neuroscience, King’s College London, London, United Kingdom; ^3^School of Computer Science and Technology, Tongji University, Shanghai, China

**Keywords:** functional MRI, action, verb, multi-voxel pattern analysis, dynamic causal modeling

## Abstract

Shared brain regions have been found for processing action and language, including the left inferior frontal gyrus (IFG), the premotor cortex (PMC), and the inferior parietal lobule (IPL). However, in the context of action and language generation that shares the same action semantics, it is unclear whether the activity patterns within the overlapping brain regions would be the same. The changes in effective connectivity affected by these activity patterns are also unclear. In this fMRI study, participants were asked to perform hand action and verb generation tasks toward object pictures. We identified shared and specific brain regions for the two tasks in the left PMC, IFG, and IPL. The mean activation level and multi-voxel pattern analysis revealed that the activity patterns in the shared sub-regions were distinct for the two tasks. The dynamic causal modeling results demonstrated that the information flows for the two tasks were different across the shared sub-regions. These results provided the first neuroimaging evidence that the action and verb generation were task context driven in the shared regions, and the distinct patterns of neural information flow across the PMC-IFG-IPL neural network were affected by the polymodal processing in the shared regions.

## Introduction

Research on the cerebral network of language and motor systems has claimed that the brain’s sensorimotor system plays an important role in language processing (Gallese and Lakoff, 2005; Binder and Desai, 2011; Glenberg and Gallese, 2012; Pulvermüller, 2013; Glenberg, 2015). Previous functional MRI (fMRI) studies have found overlapping brain regions for the generation, mental simulation, and observation of action and language processing in the premotor cortex (PMC), the inferior frontal gyrus (IFG), and the parietal cortex, especially the action and verb processing (Grezes and Decety, 2001; Hamzei et al., 2003; Baumgaertner et al., 2007; Péran et al., 2010). However, it is unclear whether action and language are processed in a “polymodal” manner within some common neural substrates, that is, whether the same brain regions are engaged in both tasks. It has been shown that the inferior frontal, parietal, and temporal–occipital cortices are activated by action semantics processing for both visually or verbally presented stimuli (Baumgaertner et al., 2007; Xu et al., 2009). This work explored whether actions and action verbs are generated in a polymodal manner in the shared brain region and investigated whether their information flows between the shared brain region were affected. The findings of this exploration may contribute to the understanding of the neuronal activity and information interaction shared by action and language generation. They may facilitate the diagnosis, evaluation, and treatment of apraxia and aphasia.

The IFG, PMC, and the inferior parietal lobule (IPL) were found to be engaged in both action and action verb generation. The PMC is engaged in both action planning and verb processing (Hauk and Pulvermüller, 2004; Baumgaertner et al., 2007). The IFG is associated with various language tasks included language generation (de Zubicaray and Piai, 2019) and semantic processing (Costafreda et al., 2006). It was also found to be engaged in action simulation and observation (Grezes and Decety, 2001). As for the IPL, it was suggested to encode action goals (Hamilton and Grafton, 2006) and action semantics in verbs (Van Dam et al., 2010). Moreover, our previous work found that the shared brain areas in the left IFG and the precentral gyrus (LIFG/PCG) showed stronger activation in a language phonological task than in an action observation task. The reverse was found in the shared areas in the left intraparietal sulcus (LIPS; Zhang et al., 2017b). Based on this, we hypothesized that action and verb generation might elicit different activity patterns in shared brain regions.

Previous studies have shown that a neural region is involved in action or language processing using univariate analysis (Hamzei et al., 2003; Péran et al., 2010; Andric et al., 2013). In comparison, the multi-voxel pattern analysis (MVPA) provides valuable means to reveal more sensitive differences in activity patterns between cognitive tasks or mental states (Haxby et al., 2001; Norman et al., 2006; Akama et al., 2012; Oosterhof et al., 2012; Zhang et al., 2017b; Sheikh et al., 2019). Using MVPA and fMRI, motor imagery and lexical decision tasks were suggested to show different activity patterns in the premotor and primary motor areas (Willems et al., 2010). Our previous study found dissociated activation patterns within shared areas in LIFG/PCG, LIPS, and the left temporal–occipital cortex between action observation and language phonology (Zhang et al., 2017b). However, the MVPA method has not been used to analyze activity patterns of action and verb generation for the same action semantics, which is one of the focuses of this study.

Analysis of the dynamic information flows between cortical regions provides another means to reveal the neural mechanism of action and verb generation. Dynamic causal modeling (DCM; Friston, 1994, 2011; Friston et al., 2003) is one of the commonly used methods to reveal the interaction between brain regions, which assumes a bilinear approximation of neural dynamics with the hemodynamic response of fMRI data. It has been widely used to investigate neural network dynamics underlying action (Sasaki et al., 2012) and language processing (Heim et al., 2009). A previous DCM study has shown that a dynamic feedback-control system involving the posterior superior temporal sulcus, the ventral premotor area, the inferior parietal lobule and the primary sensorimotor cortex is engaged in action observation (Sasaki et al., 2012). In terms of language research, the information flow between BA44 and BA45 has been found to be related to word retrieval and speech processing during word generation (Heim et al., 2009). However, DCM has not been used in analyzing the information flow among brain regions shared by action and language generation, which is another focus of the present study.

We conducted an fMRI experiment that involved both action generation and action verb generation tasks in this study. The brain regions activated by these two tasks were segmented into three sub-regions: neural areas shared by both tasks (shared sub-regions), neural areas activated only for action generation (GenA sub-regions) and those activated only for verb generation (GenV sub-regions). We hypothesized that the activity patterns in all the shared sub-regions were different between the two tasks. The difference in the activity patterns resulted in different information flows between the shared sub-regions. We tested these hypotheses by using MVPA and DCM analyses.

## Materials and Methods

### Participants

Twenty-one healthy right-handed Chinese undergraduates of Tongji University participated in this experiment (age range: 20–25 years old, 13 males, and eight females). No participant reported a history of psychiatric or neurological illness. This study was approved by the Ethics Committee in Tongji University, China. Written consent was obtained from all the participants before participation.

### Visual Stimuli

A pre-experiment was carried out to select visual stimuli from 74 candidate objects. 26, 25, and 23 objects pictures associated with each of the three actions: grasping, pressing or pinching were presented in the pre-experiment. Eleven participants performed three tasks on each object picture: first, to name the object; second, to select the most appropriate action (out of grasping, pressing or pinching) to manipulate this object; third, to score familiarity with this object (0–7, from unfamiliar to very familiar).

The goal of the pre-experiment was to select objects with high naming consistency, action consistency, and familiarity. The naming consistency and action consistency were measured by the frequency of the most frequent response divided by the frequency of the second-most frequent response. Eight object pictures were selected for each of the three actions, respectively. We initially screened object pictures with naming consistency over 10, action consistency over 4.5, and familiarity over 5. Out of these we selected the top 8 object pictures based on the action consistency related to each of the three actions.

In the end, 24 stimuli were chosen with a mean naming consistency of 10.13 (SD = 0.81), mean action consistency of 9.57 (SD = 0.24, at least 87% answers were the same), and a mean familiarity of 5.44 (SD = 0.17). The object pictures were grayscale images with a suitable size presented in a light-grey background. The visual stimuli are shown in Supplementary Figure S1.

### fMRI Acquisition and Design

All the fMRI data were acquired using a GE MR750 3T MRI scanner (GE Healthcare, Illinois, United States) in Tongji University. The following scanning parameters were used for the Echo-planar imaging (EPI) sequence: repetition time = 2,000 ms; echo time = 30 ms; field of view =220 × 220 mm^2^; 37 slices; slice thickness = 3.4375 mm; voxel size = 3 × 3 × 3.4375 mm^3^. 324 EPI images were obtained in two runs in total. T1-weighted structural images were obtained using a 3D FSPGR sequence, and the voxel size was 1 × 1 × 1 mm^3^.

This study involved three tasks under fMRI, action generation, verb generation, and object naming tasks. In the action generation task (GenA), participants were asked to generate an appropriate manipulative action to the presented object picture using their right hands. The generated action was performed without touching any object. In the verb generation task (GenV), participants were asked to generate an appropriate verb silently to a presented object picture. In the object naming task (NAM), participants silently named the object picture without any action. The NAM task was used as the baseline condition for GenA and GenV, which controlled for the processes of object picture recognition and lexical retrieval. Block designs were used for all the tasks. Rest blocks were inserted between each of the two consecutive task blocks, in which participants viewed a blank screen with a “+” sign in the center. The experimental procedure was programmed and presented using the Presentation Software v0.71 (NeuroBehavioral Systems, California, United States).

**Figure 1:**
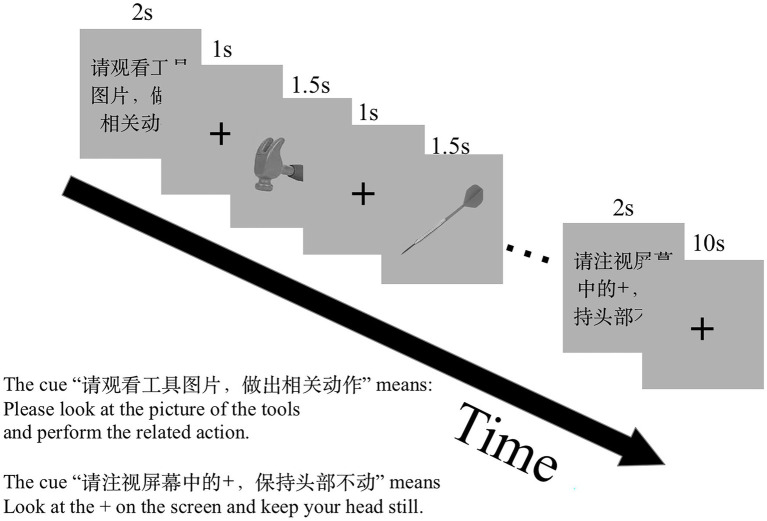
The functional MRI (fMRI) experiment paradigm design. There were two scan runs in this experiment. One run comprises three tasks: NAM, to silently name an object picture; GenA, to perform a hand gesture to an object picture; GenV, to silently generate a verb to an object picture. Before the task block, a visual cue was presented to prompt the task.

We counter-balanced the order of the tasks across participants and pseudo-randomized stimuli and block orders in each run. There were two runs in the experimental session. Twelve task blocks (four blocks for each task) and 12 rest blocks were involved in a run. A visual cue for the coming task was presented for 2 s before each task block, for example, “Please generate a verb related to the presented object silently” (Chinese, “请默读物体相关动词”) for the GenV task. Each task block involved six trials. A total of 48 trials were performed in the two runs for each of the GenA, GenV, and NAM tasks. Each trial involved a visual stimulus presented for 1,500 ms followed by a fixation cross for 1,000 ms. One rest block followed one task block. Each rest block lasted for 10 s. The whole scanning session obtained 162 volumes. The experiment design is shown in Figure 1.

It was difficult to record the behavioral data in real time under the MRI. Therefore, immediately after the scan, participants were presented with the objects and were asked to recall the actions and verbs that were generated in the experiment for each object picture. Object pictures were presented only once, in the order in which they first presented under the MRI. All stimulus images were scaled to the same size as those seen in the MRI scanner. We analyzed whether the generated responses were the same as the actions predefined in the pre-experiment. The recall task was explained to participants before the scan.

### Behavioral Analysis

For the GenA and GenV tasks, the participants’ responses in the recall task were analyzed. The accuracy was calculated by comparing the responses with the predefined actions. Moreover, paired *t*-test was employed to check if participants responded differently in the GenA and GenV for the same object.

### fMRI Data Analysis

Functional MRI data were analyzed using SPM12 (Wellcome Department of Cognitive Neurology, London, United Kingdom) implemented in MATLAB R2016B (MathWorks, Natick, MA). The BOLD images were realigned to the first slice across volumes. Head movement parameters were estimated in the realignment step. The structural images were co-registered with the mean functional images. The structural image was segmented into different tissue types: grey matter, white matter, and cerebrospinal fluid. The transformation parameters from the local space to the Montreal Neurological Institute (MNI) standard space were estimated from the segmentation step. They were used to map the functional images to the MNI space. The functional images were smoothed using an 8 mm FWHM kernel.

Two-level general linear models (GLM) were used for in the fMRI statistical analysis. At the first-level (for single participant), BOLD signals in the tasks of GenA, GenV, and NAM was modeled using GLM, comprising the onsets and durations of each task for each scanning run. The GLM model consisted of four regressors: GenA, GenV, NAM, and head movement. In this analysis, Slow signal drifts were removed by using a high-pass filter of 1/128 Hz cutoff. Serial correlations among scans were modeled using an AR(1) model, enabling maximum likelihood estimates of the whitened data. Four contrast images were derived for each participant: GenA>NAM, GenV>NAM, GenA>GenV, and GenV>GenA. Theses contrast images were subjected to a group-level analysis using one-sample *t*-tests. The activation maps were thresholded at *p* < 0.001 at the voxel level and *p* < 0.05 with family-wise error (FWE) correction at the cluster level.

We identified brain regions that were significantly activated in either GenA>NAM or GenV>NAM. Then we obtained the shared brain regions activated in contrasts using conjunction analysis. The conjunction analysis extracted the voxels that passed the statistical threshold of both contrasts. The regions in which the voxels were activated in only one task was identified as the specific region for a task. We named the shared and specific brain regions based on their anatomical locations. For example, shared regions in the left Premotor Cortex (PMC) were labeled as Shared-PMC, while the specific regions were labeled as GenA-PMC and GenV-PMC.

Then we extracted the mean activation levels of GenA>NAM and GenV>NAM in the shared regions by using nibabel.^1^ The mean activation levels of GenA>NAM were compared with those of GenV>NAM using paired *t*-test with Bonferroni correction to check if they were significantly different.

### Multi-Voxel Pattern Analysis

The Shared sub-regions were selected as regions of interest (ROIs) for MVPA. Linear support vector machines (SVMs; Cortes and Vapnik, 1995) were employed to investigate whether activity patterns could be differentiated in a shared ROI. Before classification, voxel activities were extracted from each ROI and normalized by *Z*-Score across the time and space dimension, according to Equation (1).


(1) $$u_{j}^{i}=\frac{\frac{v_{j}^{i}-\mu_{j}}{\sigma_{j}}-\mu^{i}}{\sigma^{i}}$$

$u_{j}^{i}$ is the normalized value of voxel *j* at timepoint *i*; $v_{j}^{i}$ is the original value of voxel *j* at timepoint *i*; $\mu_{j}$ and $\sigma_{j}$ are the average and standard deviation of the j th voxel timeseries; $\mu^{i}\;$ and $\sigma^{i}$ are the average and standard deviation of all voxels in the ROI at timepoint *i*. In this manner, the mean activation across voxels was normalized to zero for each task, therefore the classification performance would reflect the relative activation strengths across voxels in a ROI, rather than the ROI’s mean activation differences across tasks.

Each TR was taken as a data point for classification. There were 128 data points for the MVPA. Eight-fold cross-validation was carried out. In each fold of cross-validation, training and testing data were split by each block. In this manner, 7/8 data were involved for training and 1/8 for testing. We calculated the average classification accuracy for each subject. The primary visual cortex (V1) was selected as a control ROI. The classification accuracy of each shared region was compared with that of the V1 (Zhang et al., 2017a), using paired *t*-tests with Bonferroni correction. The MVPA analysis was implemented by sklearn 0.23.0,^2^ and the linear SVM used default hyperparameter in the sklearn toolbox.

### Dynamic Casual Modeling

We used DCM (Friston et al., 2003) implemented in the SPM12 software to analyze the information flow across brain regions. DCM is a hypothesis-driven method that incorporates related effects (e.g., task, input, and stimuli) to test the best model suitable for the experiment (Friston, 2009). DCM uses a validated biophysical model of fMRI measurements to analyze underlying information flows based on the hemodynamic response (Friston et al., 2000). Then the underlying information flow is used to estimate connectivity parameters between two connected ROIs, described in Friston et al. (2003). DCM has been widely employed to investigate the effective connectivity (Seghier et al., 2010; Sasaki et al., 2012). In this study, we performed DCM analysis on the three brain regions of the left PMC, IFG, and IPL.

**Figure 2:**
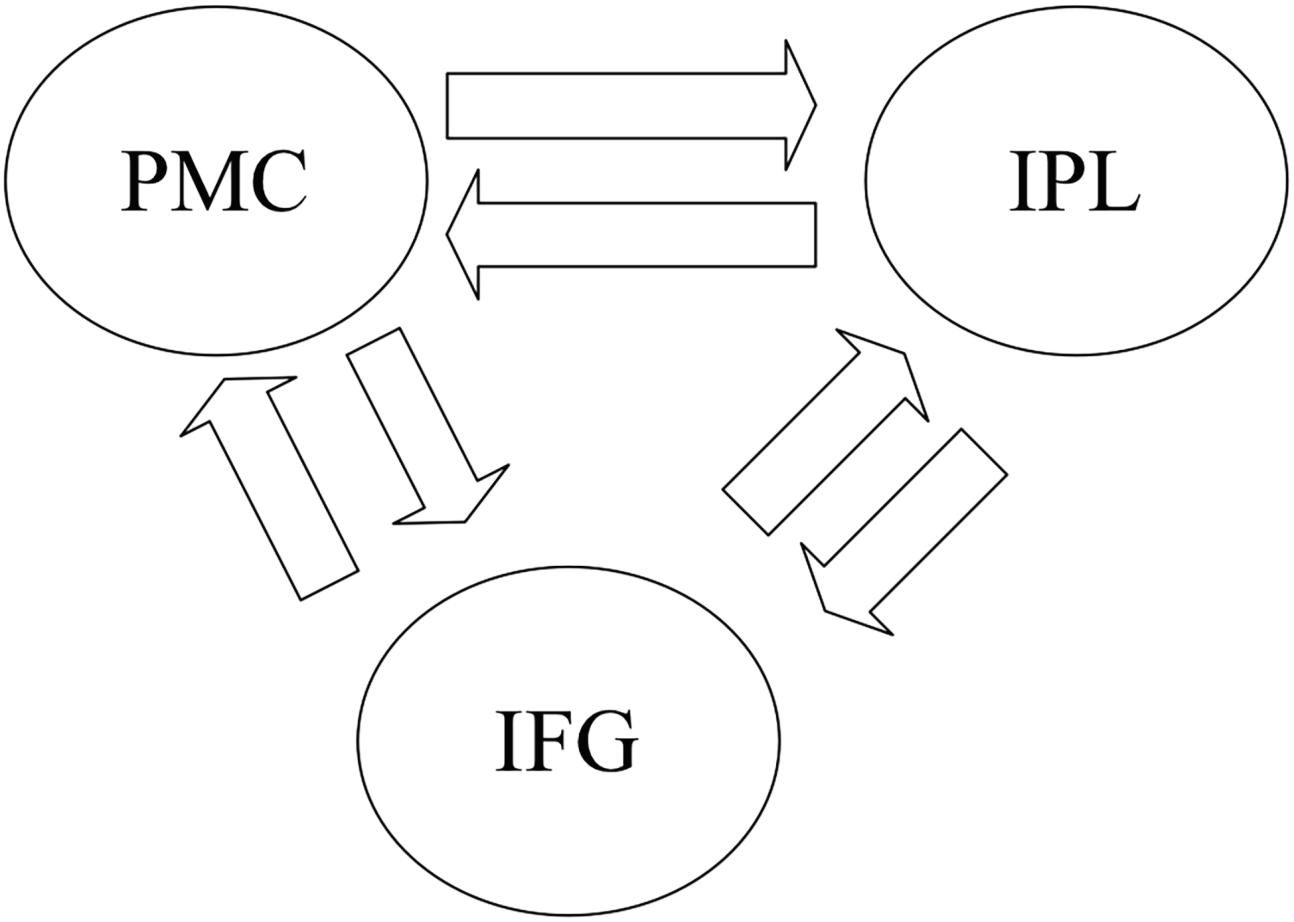
The basic dynamic causal modeling (DCM) model for the DCM across premotor cortex (PMC), inferior frontal gyrus (IFG), and inferior parietal lobule (IPL). The basic DCM model was specified with intrinsic bidirectional connections between all regions of interest (ROIs) and driving input into all Shared sub-regions in the left PMC, IFG, and IPL.

We first investigated how the information was transmitted between the shared sub-regions in the left PMC, IFG, and IPL for the GenA and GenV tasks. A basic DCM model was specified with intrinsic bidirectional connections between all ROIs and driving inputs into all ROIs (Figure 2). The basic model was then modified systematically to produce 63 alternative models with all the possible combinations of task modulatory effects. Model 1, 2, 4, 8, 16, 32 were the models with a single task modulatory effect (Supplementary Figure S2). The other models were constructed by the combinations of these six models.

In the model estimation stage, intrinsic connections and modularity effects were estimated for each subject and task, respectively. The modularity effects indicate how the source region affects the target region. The parameters of modularity effects are the linear rate of change (units of 1/s) of the target region affected by activity in the source region. Positive (or negative) modularity effects indicate that the rate of change in the target region is positively (or negatively) related to activity in the source region.

When the models at the participant level were estimated, group-level random effect Bayesian Model Selection (BMS) was performed (Seghier et al., 2010). We selected the models with the highest probability representing the most plausible models for the two tasks (GenA and GenV). Then, the estimated modulatory effects were subjected to one-sample *t*-tests to examine whether the modularity effects in the DCM model were significantly different with zero (*p* < 0.05, with Bonferroni correction). Finally, to explore which modulatory effects were affected by the task, we compared the modulatory effects of GenA and GenV on the intrinsic connections based on the optimal DCM model, by using paired *t*-tests (*p* < 0.05, with Bonferroni correction).

## Results

### Behavioral Results

The mean accuracies of GenA and GenV were both 94% (SD = 0.052, 0.053, respectively). These high accuracies showed that the responses were consistent with the predefined actions. The result of the paired *t*-test showed an insignificant difference between GenA and GenV [*t*(20) = 1.3, *p* = 0.2]. It indicated that the verbs and actions generated toward the same object were consistent.

### Brain Activation Results

**Figure 3:**
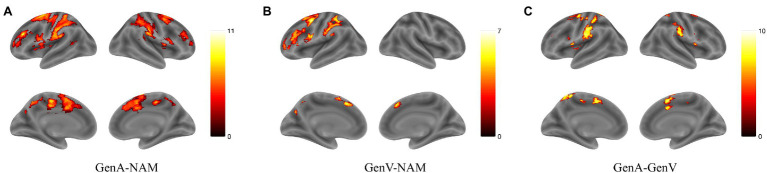
Activation maps for **(A)** GenA>NAM, **(B)** GenV>NAM, and **(C)** GenA>GenV. Color bars represent *T* values.

Activation for GenA>NAM was found in the bilateral Middle Frontal Gyrus (MFG), Superior Frontal Gyrus (SFG), inferior frontal gyrus (IFG), Middle Cingulate, the left Inferior Parietal Lobule (IPL), and the bilateral Middle Temporal Gyrus (MTG; Figure 3A; Table 1). GenV>NAM activated the Supplementary Motor Area (SMA), MFG, IFG, Precuneus, Precentral Gyrus, and SupraMarginal Gyrus on the left hemisphere (Figure 3B; Table 2).

**Table 1:** Activation peaks and extents for GenA>NAM.

GenA>NAM				MNI coordinates		
Anatomical location	Brodmann area	Voxel count	*t*-Value	*x*	*y*	*z*
Left Middle Cingulate Gyrus	6/40/4/44	3,216	11.64	−11	−23	42
Left Superior Frontal Gyrus		3,216	9.93	−25	−2	54
Left Inferior Parietal Lobule		3,216	7.86	−37	−45	45
Left Inferior Frontal Gyrus		3,216	6.51	5	−59	9
Right Middle Cingulate Gyrus	40/2/7/3	515	8.24	17	−26	45
Right SupraMarginal gyrus		515	8.20	58	−26	30
Left Middle Frontal Gyrus	46/10	245	11.42	−42	39	30
Right Inferior Frontal Gyrus	10	103	5.76	37	32	27
Right Inferior Frontal Gyrus	44	95	6.04	51	12	12
Left Middle Temporal Gyrus	39/22	39	6.73	−49	−54	9

**Table 2:** Activation peaks and extents for GenV>NAM.

GenV>NAM				MNI coordinates		
Region name	Brodmann area	Voxel count	*t*-Value	*x*	*y*	*z*
Left Precentral Gyrus	6/8/32	471	7.47	−35	1	48
Left Middle Frontal Gyrus		471	7.33	−32	−2	51
Left SMA		471	7.10	−8	18	45
Left SupraMarginal Gyrus	40/2	229	6.48	−56	−30	30
Left Inferior Parietal Lobule		229	6.46	−38	−40	48
Left Inferior Frontal Gyrus	46/9/10	263	6.07	−49	32	18
Left Inferior Frontal Gyrus	44	90	5.71	−56	8	12
Left Precuneus	7	55	4.94	−14	−68	33

GenA showed higher activation than GenV (Table 3; Figure 3C) in the bilateral postcentral gyrus, supramarginal gyrus, SMA, the left IFG, and the right superior frontal gyrus. The GenV>GenA contrast did not show significant results. From these activations, it is clear that the activation regions of GenA>NAM and GenV>NAM are located in the frontal and parietal lobes. However, the activation of the action generation task was stronger and broader than that of the verb generation.

**Table 3:** Activation peaks and extents for GenA>GenV.

GenA>GenV				MNI coordinates		
Region name	Brodmann area	Voxel count	*t*-Value	*x*	*y*	*z*
left Postcentral Gyrus	40/2	201	10.01	−56	−23	36
left SupraMarginal Gyrus		201	8.50	−49	−30	24
left SupraMarginal Gyrus	40/2	138	9.59	58	−30	27
right Postcentral Gyrus		138	7.95	44	−30	39
left SMA	6/7/32/24	581	9.44	−4	1	48
right SMA		581	9.26	3	1	54
left Rolandic Operculum	13/44	31	7.45	−45	1	15
right Superior Frontal Gyrus	6	19	7.33	27	−2	51

### Shared Sub-regions

**Figure 4:**
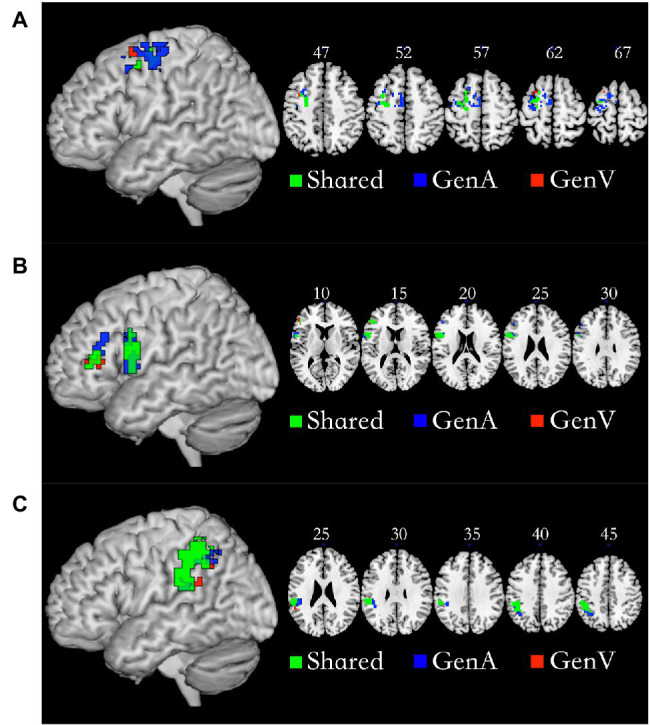
Shared and specific sub-regions in the left premotor cortex (PMC; **A**), the left inferior frontal gyrus (IFG; **B**), and the left inferior parietal lobule (IPL; **C**). **(A)** The GenA-PMC was located in the dorsal PMCd and the SMA. The GenV-PMC and Shared-PMC were located in the PMCd. **(B)** The GenA-IFG and Shared-IFG were located in Brodmann Area (BA) 44 and 45. The GenV-IFG was located in BA45. **(C)** GenA-IPL, GenV-IPL, and Shared IPL ROIs were all located in the left Supramarginal gyrus (BA40).

Conjunction analysis of GenA>NAM and GenV>NAM was employed to locate the shared regions for action generation and verb generation. The left Premotor Cortex (PMC, 92 voxels), the left IFG (103 voxels), and the left IPL (219 voxels) were activated in both tasks. The specific regions for GenA were found in GenA-PMC (149 voxels) and GenA-IFG (52 voxels), and GenA-IPL (91 voxels). The specific regions for GenV were found in GenV-PMC (six voxels) and GenV-IFG (six voxels), and GenV-IPL (six voxels). These regions are shown in Figure 4. The sub-regions of the left PMC were all located in Brodmann Area 6. The GenA-PMC was located in the dorsal PMC (PMCd) and the supplementary motor area (SMA). The GenV-PMC and Shared-PMC were located in the PMCd. The GenA-IFG and Shared-IFG were located in BA 44 and 45. However, the GenV-IFG was only found located in BA 45. All of the sub-regions of IPL were located in the left Supramarginal gyrus (BA40).

### Mean Activation in the Shared Areas

**Figure 5:**
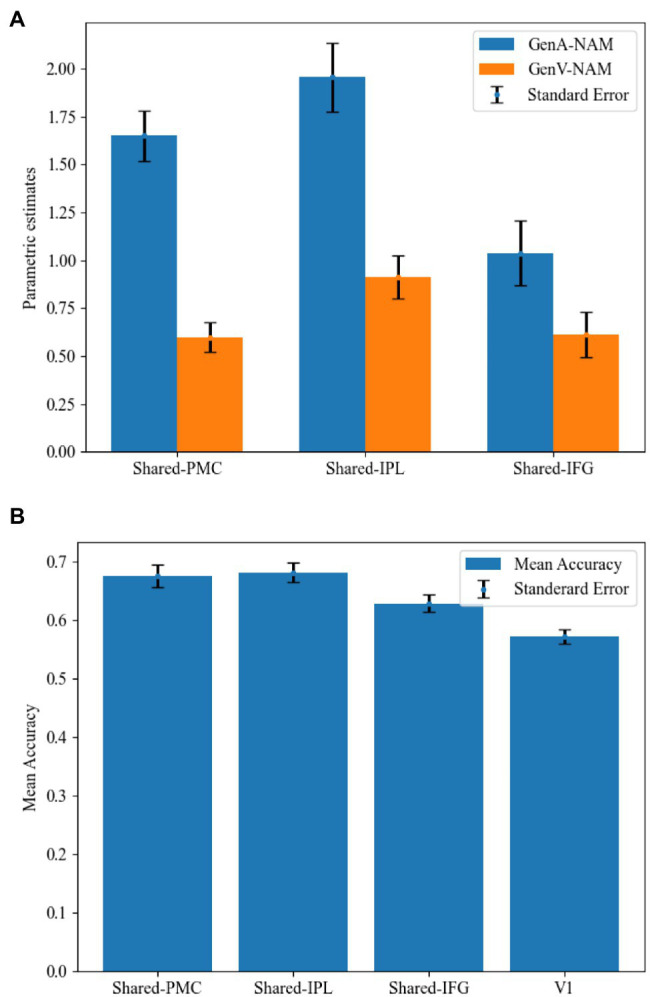
Comparisons between the GenA and GenV tasks in the shared regions. **(A)** Estimated parameters for GenA-NAM and GenV-NAM conditions. **(B)** The classification accuracy in each shared region and the V1.

Mean activation levels were compared between GenA and GenV tasks in all the Shared areas. Mean activation levels for GenA was significantly higher than GenV in Shared-PMC [*t*(21) = 8.39, *p* < 0.001], Shared IPL [*t*(21) = 7.74, *p* < 0.001], and Shared-IFG [*t*(21) = 4.10, *p* < 0.001]. The comparison results showed that the activation intensity of action generation was higher than that of verb generation in all three shared regions. Activation estimates for GenA and GenV (NAM as the control condition) are shown in Figure 5A.

### MVPA Results

In the MVPA, the activity patterns of GenA and GenV tasks were classified using a linear SVM classifier. Classification accuracies in Shared-PMC [mean = 67.60%, SE = 0.019, *t*(21) = 4.33, *p* < 0.001], Shared IPL [mean = 68.20%, SE = 0.017, *t*(21) = 5.27, *p* < 0.001], and Shared-IFG [mean = 62.90%, SE = 0.015, *t*(21) = 2.71, *p* = 0.007] were found to be significantly greater than that in the control ROI of V1 (mean = 57.20%, SE = 0.012). MVPA results are shown in Figure 5B. Given that the mean activation level difference has been removed in normalizing the activity patterns, this result indicated that the relative action strengths across voxels were different between GenA and GenV.

### DCM Across PMC, IFG, and IPL

**Figure 6:**
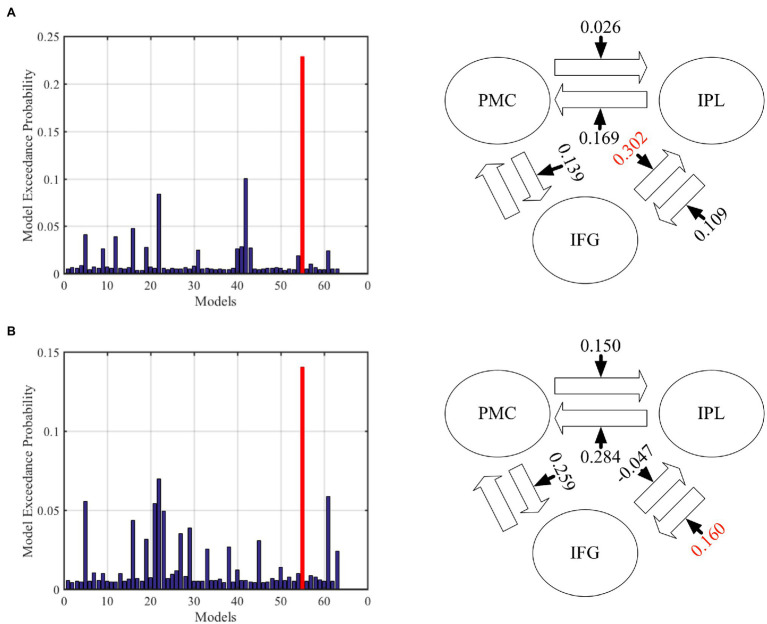
Results of dynamic causal modeling (DCM) analysis across premotor cortex (PMC), inferior frontal gyrus (IFG), and inferior parietal lobule (IPL). **(A)** The best-fit DCM model with GenA modulatory effect. **(B)** The best-fit DCM model with GenV modulatory effect. The numbers on the solid arrows indicate the average modulatory effects. The numbers in red indicate that the modulatory effects survive Bonferroni correction for multiple comparisons.

In the DCM analysis for the GenA and GenV tasks, model 55 resulted in the highest model fitting (Figure 6; Supplementary Figure S2). In this best-fit model, the GenA effect was significantly above zero for IFG → IPL connection [mean = 0.302, *t*(21) = 2.716, *p* = 0.010], and the GenV effect was significantly above zero for the IPL → IFG connection [mean = 0.160, *t*(21) = 2.700, *p* = 0.010], shown in Figure 4. The GenV effects on three more connections were higher than 0, but did not passed the Bonferroni correction: PMC → IPL [mean = 0.284, *t*(21) = 2.151, *p* = 0.037] and PMC → IFG [mean = 0.259, *t*(21) = 2.271, *p* = 0.028]. Paired *t*-tests were used to compare the modulatory effects of GenA and GenV on the same connection. It was found that the modulatory effect of GenA was significantly higher than that of GenV on the IFG → IPL connection [*t*(21) = 1.822, *p* = 0.038], while the opposite effect was found on the PMC → IPL connection.

## Discussion

This study found shared and specific sub-regions in the left IFG, premotor cortex, and inferior parietal lobule on action generation and verb generation tasks. However, the mean activation level and activity patterns in the shared sub-regions were shown to be significantly different. These results suggest that although the same groups of voxels are activated by the generation of actions and verbs, they were engaged in the two tasks differently. Finally, we found that the task modulatory effects on specific connections in the information flow networks were significantly different. From the above results, we can speculate that the different task modulatory effects may result from the separation of the activity patterns of voxels in the shared activated sub-regions.

### Shared and Specific Brain Sub-regions for Action and Verb Generation

The results of conjunction analysis suggest that the left IFG, premotor cortex and inferior parietal lobule are common neural substrates for action and verb generation. The left PMC and left IFG have been found to play a role in action observation and action-related language understanding (Hamzei et al., 2003; Desmurget and Sirigu, 2009; Zhang et al., 2017b, 2018; Maranesi et al., 2019; Jerjian et al., 2020). The left IPL is suggested to be engaged in action coding, peripersonal space representation, action intention understanding, and action verb processing (Fogassi et al., 2005; Fogassi and Luppino, 2005; Van Dam et al., 2010). Our results further suggest that the left IFG, premotor cortex, and inferior parietal lobule are shared by action and action verb generation.

Moreover, we identified neural sub-regions activated for either action generation (GenA) or verb generation (GenV) in the three brain regions. The brain region-specific GenA and GenV were found located in the PMCd. The previous research has consistently found the activation of left PMCd for action execution and verb generation (Grafton et al., 1997; Grezes and Decety, 2001). Parts of sub-regions specific to GenA were also found in the left SMA, which is consistent with the findings of previous research (Goldberg, 1985). Previous studies have found that both the covert verb generation (Péran et al., 2010; Allendorfer et al., 2012) and overt verb generation (Alario et al., 2006; Allendorfer et al., 2012) were activated in the SMA. In our results the covert verb generation task also activated the SMA. Furthermore, shared brain areas in the SMA was also activated in action generation. It implies that SMA can process the action concept encoding involved in action and verb generation. In the left IFG, the sub-region specific to GenA was found located in BA 44 and 45, while the sub-region specific to GenV was only found located in BA 45. These results corroborate previous findings that the left BA 45 is related to lexical search (Heim et al., 2005). In addition, we also found that the left Broca’s area (BA 44/45) was involved in action generation. The shared and specific sub-regions of IPL were found located in the left Supramarginal gyrus (BA40). The Supramarginal gyrus has been suggested to play a role in verbal working memory (Deschamps et al., 2014) and finger positioning for object use (Andres et al., 2017), which were involved in the GenV and GenA tasks, respectively.

### Differentiable Neural Activity Within the Shared Areas

The mean activation level comparisons showed that all the shared sub-regions were more significantly activated for GenA than for GenV. According to the behavioral results (in Section “Behavioral Results”), the semantics of generated actions and verbs were similar. Therefore, the activated regions of GenA and GenV processed action and verb retrieval and generation cognitive processes with the same semantics. Although the actions and verbs have the same semantics, in the GenA task, the actions were actually performed. In the GenV task, the verbs were silently generated. The difference in activation levels could be caused by how the tasks were performed (overt vs. covert). The overt generation task could cause stronger neural activities, as shown in a previous study (Forn et al., 2008). Therefore, the differences in mean activation level could be due to the difference in the task goal (action vs. verb), and/or the difference in the operation mode (overt vs. covert), which cannot be teased apart with our experimental design and is a limitation of this study.

The MVPA demonstrated that the normalized voxel activity of each shared ROI in the left PMC, IFG, and IPL was also different for GenA and GenV. These results suggest that the shared regions process action semantic information and are modulated by the task context for how action semantic is generated. And according to the mean activation level analysis and MVPA (Section “Mean Activation in the Shared Areas” and “MVPA Results”), it could be found that the difference in the Shared-IFG was lower than those in the other two shared sub-regions. The IFG is well known to be an essential region for language generation. Studies have found that parts of IFG were also involved in the actual generation or execution of actions (Grezes and Decety, 2001; Péran et al., 2010). The results of mean action level analysis and MVPA support the involvement of the left IFG for action generation. However, it is less involved in the left IFG than the left PMC and IPL. The PMC and IPL were deemed pivotal regions for action execution (Grezes and Decety, 2001; Fogassi et al., 2005).

These results corroborate a previous study showing that the neural activity within shared regions are action-specific or phonology-specific according to the task context (Zhang et al., 2017b). Moreover, Brodmann’s area 44 (located in the IFG) was found to be endowed with polymodal capabilities (Binkofski and Buccino, 2006; Baumgaertner et al., 2007). We extended these findings by revealing polymodal processing for the action and verb generation not only in the IFG, but also in the PMC and IPL.

### Information Flow Between Shared Regions

In this study, DCM was employed to investigate the information flow among shared regions for action and verb generation to reveal the underlying mechanisms of polymodal processing. The results showed that although the same model (Model 55) was the best-fit model for both GenA and GenV, there were significant differences in the task modulatory effects between the two tasks. In the GenA and GenV models, the task modulation effects on IFG → IPL and IPL → IFG connections were both significantly above zero (Table 4). By comparing the task modulation effects between the two models, it was found that the effect of GenA on IFG → IPL was significantly higher than that of GenV. In contrast, the opposite was found for PMC → IPL (Table 5).

**Table 4:** Results of one-sample *t*-test for modulatory effects for the winning model (No. 55).

Task	Connection	Modulatory effect	*t*-Value	*p*-Value
GenA	IPL → PMC	0.169 (0.84)	1.307	0.198
	PMC → IPL	0.026 (0.54)	0.313	0.756
	IFG → IPL	0.302 (0.72)	2.716	0.010^*^^,^^†^
	IPL → IFG	0.109 (0.61)	1.165	0.251
	PMC → IFG	0.139 (0.79)	1.139	0.261
GenV	IPL → PMC	0.150 (0.45)	1.310	0.198
	PMC → IPL	0.284 (0.86)	2.151	0.037^*^
	IFG → IPL	−0.047 (0.86)	−0.353	0.726
	IPL → IFG	0.160 (0.38)	2.700	0.010^*^^,^^†^
	PMC → IFG	0.259 (0.74)	2.271	0.028^*^

All ROIs are shared sub-regions.

* The modulatory effect is significantly different with 0 (*p* < 0.05).

† The *p* value survives Bonferroni correction for five tests.

**Table 5:** Results of paired-sample *t*-test for comparing the modulatory effects in GenA vs. GenV.

Connection	*t*-Value	*p*-Value
IPL → PMC	0.135	0.447
PMC → IPL	−1.713	0.047^*^
IFG → IPL	1.822	0.038^*^
IPL → IFG	−0.490	0.687
PMC → IFG	−0.777	0.779

* Means modulatory effects were significantly different with each other (*p* < 0.05).

The left PMC, IFG, and IPL were suggested to be parts of the mirror neuron system. The mirror neurons were found to be activated in observing and executing actions (Rizzolatti and Craighero, 2004; Cattaneo and Rizzolatti, 2009), and also in processing action-related language (Aziz-Zadeh et al., 2006; Fogassi and Ferrari, 2007). We suggest that the mirror neuron activity might play a role that results in the differences in the task modulation effects. The IFG → IPL connectivity may play a role in linking action goals coded in IFG to action specifications coded in IPL, which was reflected in the higher modulation effect under action generation. On the other hand, the stronger modulation effect of verb generation on the PMC → IPL connectivity may indicate that the mirror neurons encoding potential actions in the left PMC were activated and then passed on to the left IPL for further semantic processing/selection (Karunanayaka et al., 2010).

The above results suggest that neural activities within shared sub-regions in the left PMC, IFG, and IPL are modulated by the top-down cognitive requirements. These results corroborate findings from our previous study (Zhang et al., 2017b) that the shared regions demonstrate distinct activity patterns driven by tasks demands. We further demonstrated that the polymodal processing undertaken by the shared neural substrates within the left PMC, IFG, and IPL affected neural information flows between these regions.

### Limitations of This Work

We noted two limitations of this study. The first one is that in the fMRI experiment, the action was overtly generated while the verb was covertly generated. Therefore, the differences between the neural activities underlying action generation and verb generation tasks may partly be caused by whether the task is overtly or covertly performed. A covert verb generation was chosen in order to reduce head motion caused by mouth/tough movement. The reason why overt verb generation was selected is that covert action generation (motor imagery) is likely to be confused with verb generation for our participants. Therefore, in this work, the different neural activities between action and verb generation might be partly due to whether the task was overt performed or not.

The second limitation was the lack of behavior data collection under fMRI. Due to the lack of effective behavioral feedback recording methods, the experiment could not record participants’ actions and silently generated verbs in real time. Therefore, we asked participants to recall the generated actions and verbs immediately after the MRI scanning according to the stimuli images with the same size as they viewed the MRI scanner. Although this method can accurately record participants’ behavioral responses, it is difficult to exclude the trail records with inconsistent behaviors due to the lack of specific feedback record for each trail, which could ensure that participants’ feedback intentions in actions and verb generation tasks are exactly the same. Therefore, although there is no statistically significant difference between the generated actions and verbs in this experiment, it might also cause a small impact on the experimental results.

## Conclusion

This fMRI study identified shared and specific brain regions for action and verb generation tasks in the left PMC, IFG, and IPL. The mean activation level analysis and MVPA revealed different activity patterns of the shared sub-regions with various degrees in the two tasks. Finally, the dynamic causal modeling modulatory effects on IFG → IPL and PMC → IPL were significantly different. We provided a potential explanation that the backward stimulation of mirror neurons elicited by action and verb processing resulted in the different modulations. Due to the limitation of the experiment, the operation mode (overt vs. covert) may also have caused the neural activity differences between action and verb generation, which needs future investigation in future studies. Our results suggest that the top-down task demands modulate the neural activities in the shared regions of action and verb generation and that different neural information flows in PMC-IFG-IPL neural networks are influenced by polymodal processing within the shared neural regions.

## Data Availability Statement

The raw data supporting the conclusions of this article will be made available by the authors, without undue reservation.

## Ethics Statement

The studies involving human participants were reviewed and approved by Ethics Committee in Tongji University. The patients/participants provided their written informed consent to participate in this study.

## Author Contributions

ZW contributed to the writing, data processing, and data collection of this work. ZZ contributed to the paper revision, experimental design, and result analysis of this work. YS provided the main idea of this work and all resources for the experiment. All authors contributed to the article and approved the submitted version.

## Funding

This research is supported by the Fundamental Research Funds for the Central Universities (2232021D-26).

## Conflict of Interest

The authors declare that the research was conducted in the absence of any commercial or financial relationships that could be construed as a potential conflict of interest.

## Publisher’s Note

All claims expressed in this article are solely those of the authors and do not necessarily represent those of their affiliated organizations, or those of the publisher, the editors and the reviewers. Any product that may be evaluated in this article, or claim that may be made by its manufacturer, is not guaranteed or endorsed by the publisher.

## Footnotes

^1^ https://nipy.org/nibabel/

^2^ https://scikit-learn.org/

## Supplementary Material

Supplementary Figure S1 Visual stimuli in this experiment.

Supplementary Figure S2 Predefined DCM models across PMC, IFG, and IPL. Model 1, 2, 4, 8, 16, 32 were the basic models with single task effect. The additional 57 models are the combinations of these six models. “+” means the combination of two models. Model 3 = 1 + 2, Model 5 = 1 + 4, Model 6 = 2 + 4, Model 7 = 1 + 2 + 4, Model 9 = 1 + 8, Model 10 = 2 + 8, Model 11 = 1 + 2 + 8, Model 12 = 4 + 8, Model 13 = 1 + 4 + 8, Model 14 = 2 + 4 + 8, Model 15 = 1 + 2 + 4 + 8, Model 17 = 1 + 16, Model 18 = 2 + 16, Model 19 = 1 + 2 + 16, Model 20 = 4 + 16, Model 21 = 1 + 4 + 16, Model 22 = 2 + 4 + 16, Model 23 = 1 + 2 + 4 + 16, Model 24 = 8 + 16, Model 25 = 1 + 8 + 16, Model 26 = 2 + 8 + 16, Model 27 = 1 + 2 + 8 + 16, Model 28 = 4 + 8 + 16, Model 29 = 1 + 4 + 8 + 16, Model 30 = 2 + 4 + 8 + 16, Model 31 = 1 + 2 + 4 + 8 + 16, Model 33 = 1 + 32, Model 34 = 2 + 32, Model 35 = 1 + 2 + 32, Model 36 = 4 + 32, Model 37 = 1 + 4 + 32, Model 38 = 2 + 4 + 32, Model 39 = 1 + 2 + 4 + 32, Model 40 = 8 + 32, Model 41 = 1 + 8 + 32, Model 42 = 2 + 8 + 32, Model 43 = 1 + 2 + 8 + 32, Model 44 = 4 + 8 + 32, Model 45 = 1 + 4 + 8 + 32, Model 46 = 2 + 4 + 8 + 32, Model 47 = 1 + 2 + 4 + 8 + 32, Model 48 = 16 + 32, Model 49 = 1 + 16 + 32, Model 50 = 2 + 16 + 32, Model 51 = 1 + 2 + 16 + 32, Model 52 = 4 + 16 + 32, Model 53 = 1 + 4 + 16 + 32, Model 54 = 2 + 4 + 16 + 32, Model 55 = 1 + 2 + 4 + 16 + 32, Model 56 = 8 + 16 + 32, Model 57 = 1 + 8 + 16 + 32, Model 58 = 2 + 8 + 16 + 32, Model 59 = 1 + 2 + 8 + 16 + 32, Model 60 = 4 + 8 + 16 + 32, Model 61 = 1 + 4 + 8 + 16 + 32, Model 62 = 2 + 4 + 8 + 16 + 32, Model 63 = 1 + 2 + 4 + 8 + 16 + 32.
