# Supplementary figures and images for: Different Neural Information Flows Affected by Activity Patterns for Action and Verb Generation
Source: Front Psychol. 2022 Mar 24;13:802756. doi: 10.3389/fpsyg.2022.802756 (PMC8987928; doi:10.3389/fpsyg.2022.802756)

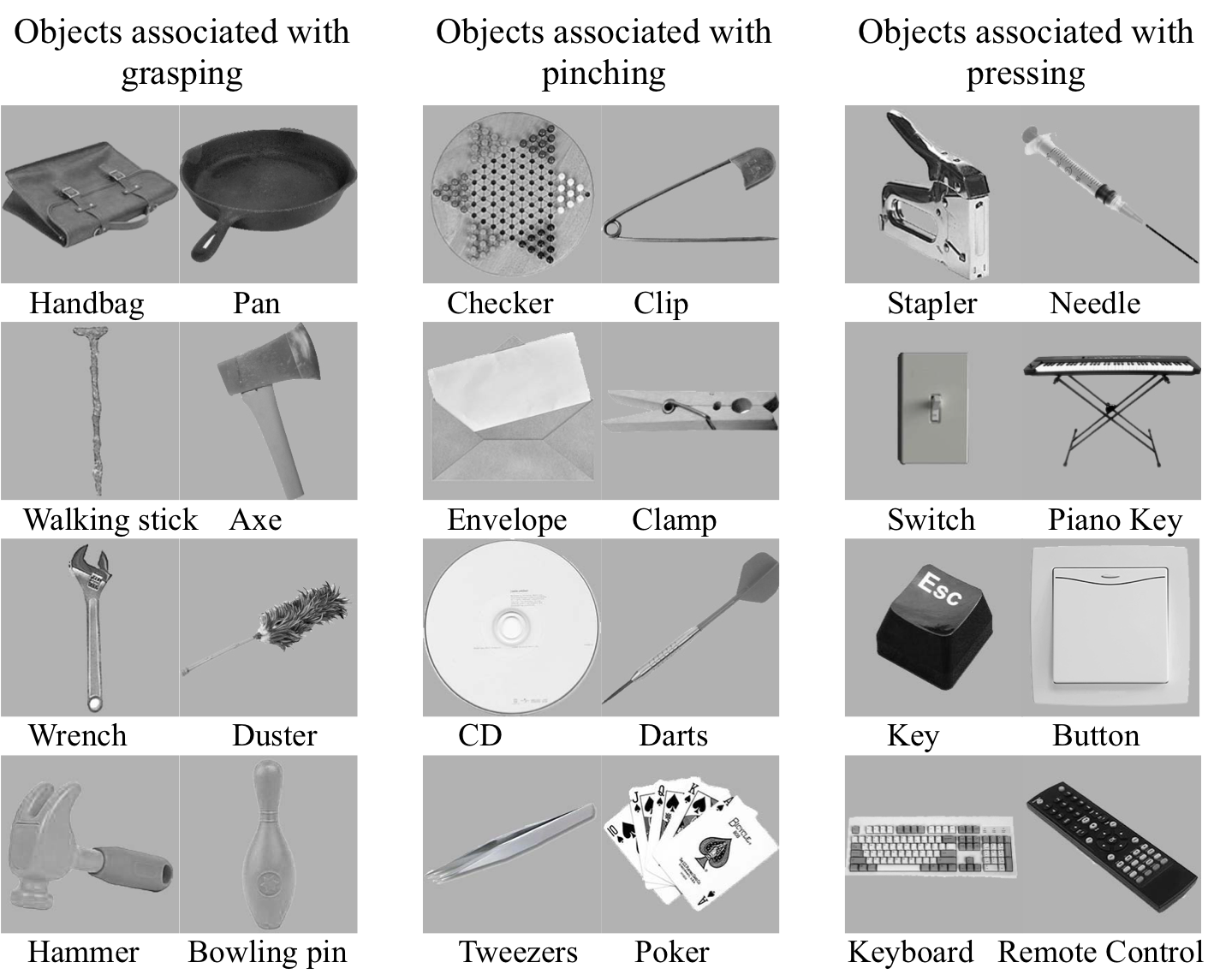

Supplement: Supplementary Figure S1 — Visual stimuli in this experiment. [file Image_1.TIFF]

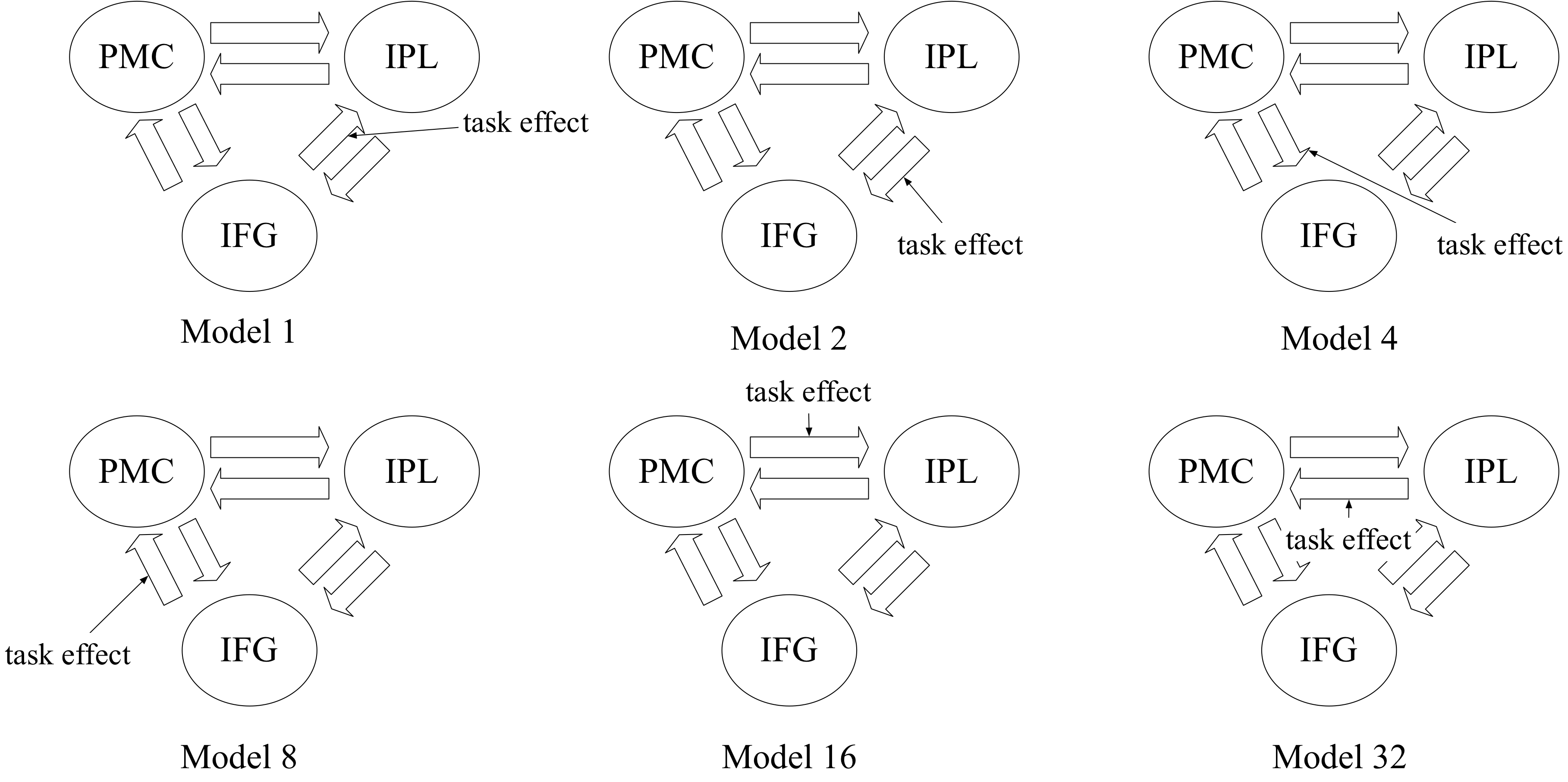

Supplement: Supplementary Figure S2 — Predefined DCM models across PMC, IFG, and IPL. Model 1, 2, 4, 8, 16, 32 were the basic models with single task effect. The additional 57 models are the combinations of these six models. “+” means the combination of two models. Model 3 = 1 + 2, Model 5 = 1 + 4, Model 6 = 2 + 4, Model 7 = 1 + 2 + 4, Model 9 = 1 + 8, Model 10 = 2 + 8, Model 11 = 1 + 2 + 8, Model 12 = 4 + 8, Model 13 = 1 + 4 + 8, Model 14 = 2 + 4 + 8, Model 15 = 1 + 2 + 4 + 8, Model 17 = 1 + 16, Model 18 = 2 + 16, Model 19 = 1 + 2 + 16, Model 20 = 4 + 16, Model 21 = 1 + 4 + 16, Model 22 = 2 + 4 + 16, Model 23 = 1 + 2 + 4 + 16, Model 24 = 8 + 16, Model 25 = 1 + 8 + 16, Model 26 = 2 + 8 + 16, Model 27 = 1 + 2 + 8 + 16, Model 28 = 4 + 8 + 16, Model 29 = 1 + 4 + 8 + 16, Model 30 = 2 + 4 + 8 + 16, Model 31 = 1 + 2 + 4 + 8 + 16, Model 33 = 1 + 32, Model 34 = 2 + 32, Model 35 = 1 + 2 + 32, Model 36 = 4 + 32, Model 37 = 1 + 4 + 32, Model 38 = 2 + 4 + 32, Model 39 = 1 + 2 + 4 + 32, Model 40 = 8 + 32, Model 41 = 1 + 8 + 32, Model 42 = 2 + 8 + 32, Model 43 = 1 + 2 + 8 + 32, Model 44 = 4 + 8 + 32, Model 45 = 1 + 4 + 8 + 32, Model 46 = 2 + 4 + 8 + 32, Model 47 = 1 + 2 + 4 + 8 + 32, Model 48 = 16 + 32, Model 49 = 1 + 16 + 32, Model 50 = 2 + 16 + 32, Model 51 = 1 + 2 + 16 + 32, Model 52 = 4 + 16 + 32, Model 53 = 1 + 4 + 16 + 32, Model 54 = 2 + 4 + 16 + 32, Model 55 = 1 + 2 + 4 + 16 + 32, Model 56 = 8 + 16 + 32, Model 57 = 1 + 8 + 16 + 32, Model 58 = 2 + 8 + 16 + 32, Model 59 = 1 + 2 + 8 + 16 + 32, Model 60 = 4 + 8 + 16 + 32, Model 61 = 1 + 4 + 8 + 16 + 32, Model 62 = 2 + 4 + 8 + 16 + 32, Model 63 = 1 + 2 + 4 + 8 + 16 + 32. [file Image_2.TIFF]
